# *Saccharomyces bayanus* Enhances Volatile Profile of Apple Brandies

**DOI:** 10.3390/molecules25143127

**Published:** 2020-07-08

**Authors:** Magdalena Januszek, Paweł Satora, Łukasz Wajda, Tomasz Tarko

**Affiliations:** 1 Department of Fermentation Technology and Microbiology, Faculty of Food Technology, University of Agriculture in Krakow, Balicka Street 122, 30-149 Kraków, Poland; pawel.satora@urk.edu.pl (P.S.); tomasz.tarko@urk.edu.pl (T.T.); 2 CDC Poland Sp. z o.o., ul. Zagnańska 153, 25-563 Kielce, Poland; lukasz.wajda8@gmail.com

**Keywords:** apple brandies, volatile compounds, terpenes, esters, higher alcohols, spontaneous fermentation, *Saccharomyces cerevisiae*, *Saccharomyces bayanus*

## Abstract

Qualitative and quantitative profiles of volatiles in alcoholic beverages depend mainly on the quality of raw materials, yeasts used for fermentation, and processing technique. *Saccharomyces bayanus* is a yeast species which is not commonly used for the production of alcoholic beverages, but it is able to produce volatiles that add desirable aroma. Since there is little information regarding the application of that microorganism for the production of apple brandies and how it affects volatile profile of finished products, we decided to address that issue. The aim of the study was to determine the impact of *S. bayanus* on the profile of volatile compounds and sensory properties of apple spirits obtained from three apple cultivars (Topaz, Rubin, and Elise) in comparison to spirits obtained from fermentation carried out spontaneously or with *Saccharomyces cerevisiae*. Obtained brandies were analysed using gas chromatography–flame ionization detector (GC–FID), solid phase microextraction–gas chromatography–mass spectrometry (SPME–GC–MS) and sensorially. In our study, brandies produced from musts fermented by *S. bayanus* demonstrated the highest concentration of ethyl esters and increased concentrations of isoamyl acetate, 2-phenylethyl acetate, ethyl palmitate and hexanol. Moreover, our results support the hypothesis that non-*Saccharomyces* yeasts which are present during spontaneous fermentation and demonstrate higher β-glucosidase activities enhance aroma of alcoholic beverages through releasing aroma compounds from glycosidic forms, e.g., α-phellandrene, (*E*)-β-fanesene, (*Z*,*E*)-α-farnesene, α-farnesene, and farnesol. Considering results obtained in sensory analysis, we proved that *S. bayanus* is suitable for the production of apple brandies, improving their flavour. Brandies obtained from musts fermented by *S. bayanus* obtained the highest average range for “overall note” parameter in sensory analysis.

## 1. Introduction

Fruit distillates are valued worldwide due to their unique flavour. Fruit distillates (called brandies) are produced by alcoholic fermentation and distillation of fleshy fruit or must of those fruits, berry or vegetable, with or without stones, and ethanol content obtained after distillation is less than 86% [1]. Substances responsible for the aroma could be divided into two groups: the first group consists of substances that originate from raw materials, and the second group consists of substances that are formed during fermentation, distillation, and maturation. Qualitative and quantitative profiles of volatiles in fruit distillates are variable [2]. The specific apple brandy calvados is under appellation d’origine contrôlée (AOC) and it is an aged product manufactured from fruits that belong to particular types of apple: sweet, tart, and bitter. Apple brandies contain numerous hexenyl esters, norisoprenoidic derivatives, unsaturated alcohols, and aldehydes. Characteristic compounds in apple spirits are ethyl acetate, ethyl lactate, ethyl succinate, 2-butanol, 4-ethylguaiacol, eugenol, and 2-propen-1-ol [1].

The origin of volatile compounds in apple alcoholic beverages is very diverse. Parts of the volatiles are already present in fruits. Some of the components, which contribute to the flavour of alcoholic beverages, originate from the raw material but are released/transformed throughout fermentation by microorganisms [3]. Numerous aroma compounds are also produced by yeast during fermentation, including higher alcohols, esters, volatile acids, carbonyl compounds, and many others.

Traditionally, fermentation is carried out by monoculture of yeast, which provides consistency in the aroma profile and relatively high content of ethyl alcohol. However, to obtain characteristic flavours, increased concentration of desirable esters, and added aroma complexity, winemakers turn to non-*Saccharomyces* species [4] or strains that belong to the *Saccharomyces* genus other than *S. cerevisiae* [5]. It must be highlighted that there are several commercially available strains that belong to both these groups [6,7]. In many cases, indigenous microbiota provides wines with desirable flavour, intense aroma persistency, distinction, and vintage variation [8]. Most often, spontaneous fermentation is started by non-*Saccharomyces* yeast (*Hanseniaspora/Kloeckera*, *Metschnikowia*, *Candida*) and finished by wild cultures of *Saccharomyces* [9].

*Saccharomyces bayanus* is a yeast species used for fermentation, particularly in winemaking [10]. Despite that it belongs to the *Saccharomyces* genus and shows genetic similarity to other species that belong to this taxon, they may vary in terms of oenological properties and the ability to synthesise volatile compounds [11]. A comparison made between *S. bayanus* and *S. cerevisiae* showed that wines fermented by *S. bayanus* have higher flavour intensity [10]. *S. bayanus* produces larger amounts of 2-phenylethanol, ethyl lactate, 2-phenylethyl acetate, and other acetate esters [10,12], while *S. cerevisiae* synthesises more isobutanol, isoamyl alcohol, and amyl alcohol [10]. Therefore, in the current study, we decided to examine profiles of volatile compounds of apple brandies obtained after fermentation of apple musts with *S. bayanus*. We compared obtained results to those obtained in the same set of experiments for spontaneous fermentation and fermentation carried out by *S. cerevisiae*. The strain of *S. cerevisiae* (Ethanol Red) used in the current study was also applied in our previous research [13] because it produced high-quality brandies. *S. bayanus* has not been used for the production of brandies from common dessert apples in Poland; therefore, we decided to examine its strain in the current study. It is expected that spontaneous fermentation should provide the most diverse profile of volatile compounds, so we tested alcoholic beverages obtained after fermentation carried out with native apple microbiota for comparison. Three apple cultivars (Topaz, Rubin, and Elise) used in experiments were chosen out of 10 cultivars after preliminary studies [13] because they demonstrated best oenological characteristics out of 10 cultivars tested. Selected apples demonstrated the best features for brandies production (high content of total extract and total sugars, high concentrations of nitrogen compounds, and suitable acidity). Moreover, these apple cultivars are commonly used for processing in the food industry, including fermentation.

The main goal of the current study was to determine the impact of different types of fermentation (spontaneous vs. *S. cerevisiae* vs. *S. bayanus*) on the volatile profile and sensory properties of apple brandies.

## 2. Results and Discussion

### 2.1. Fermentation and Chemical Composition of Fresh and Fermented Apple Musts

**Figure 1:**
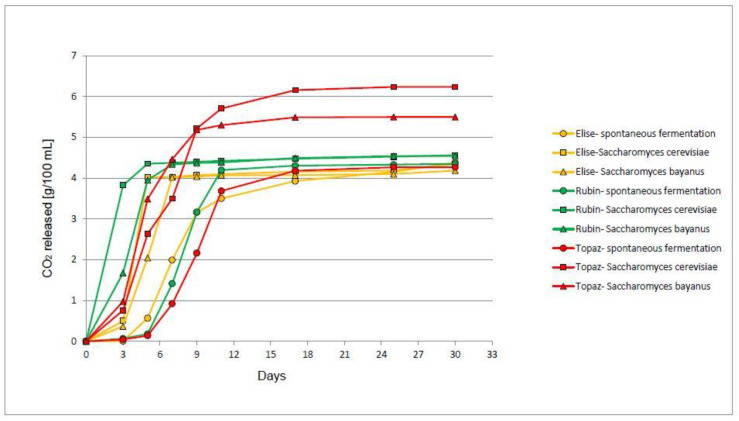
Fermentation dynamics of apple musts fermented with different type of yeasts, *n* = 5, STD < 5%. Abbreviations: SF—spontaneous fermentation, SC—fermentation with *Saccharomyces cerevisiae* (Ethanol Red), SB—fermentation with *Saccharomyces bayanus* (cider yeast).

Fermentation kinetics were studied based on weight loss associated with the liberation of carbon dioxide. Fermented musts obtained from the Topaz cultivar demonstrated the highest weight loss (Figure 1). This could be attributed to the fact that this cultivar contained significantly more total extract (Table 1) and glucose (Table 2) than others, and it is the first carbon source used in fermentation. It might be stated that the Topaz cultivar demonstrated a chemical composition that enables initiation of fermentation faster than Elise or Rubin, e.g., with regard to the profile of amino acids or organic acids. However, this hypothesis should be verified in further studies. Moreover, Elise demonstrated the lowest total extract (97 g/L), concentration of sugar, titratable acidity, and free amino nitrogen content of all analysed samples (Table 1), which may suggest that fruits were harvested before reaching consumption maturity [14]. Therefore, we should not exclude the application of that apple cultivar for the production of brandies.

In apples, fructose is the predominant sugar [14], and in the case of the analysed samples, this carbohydrate was present in the highest concentration—above 60% of total sugars (Table 2). In addition to fructose, apples also contain glucose and sucrose, and the relative amounts of these sugars differ significantly between apple cultivars. The sugar profiles of raw materials may affect the fermentation efficiency and concentration of residual sugar [14] as it happened in our study—most of the sugars were utilised during fermentation, in fermented musts resulting in small amounts of glucose (from 0.03 to 0.68 g/L) and fructose (from 0.87 to 3.24 g/L; Table 2). The concentration of glucose after fermentation was approximately the same in all samples since it is used first during fermentation. Fructose is used after glucose [15], and since its level was higher in Topaz in unfermented must (Table 2), more of that sugar remained after fermentation.

It has also been shown that the use of a different microorganism affected the rate of fermentation (Figure 1) because for each tested apple cultivar (Topaz, Elise, and Rubin), the equilibrium was reached in the shortest time when fermentation was carried out by *S. cerevisiae*, then *S. bayanus*, and the longest period of adaptation and slower fermentation were found in musts fermented spontaneously. The turbulent stage of fermentation in these samples began only after the third day and it had a negative impact on the final weight loss of these samples. Moreover, the slowdown of fermentation might be profitable due to lesser heat release, smaller tendency for excessive temperature increase during fermentation [16], and increased formation of terpenes. That slowdown of spontaneous fermentation could be caused by the shift in fungal microbiota, because as fermentation progresses, non-*Saccharomyces* species successively die off and *S. cerevisiae* begins to dominate and completes the fermentation [9].

Tarko et al. (2018) [16] showed that the fermentation process was slowest in case of must inoculated with *S. bayanus* in comparison to other yeast like in our study. We might suppose that the explanation for that phenomenon is that *S. bayanus* uses sugars for the synthesis of volatile compounds with high boiling temperature rather than carbon dioxide or small molecules of volatiles that could be easily lost during fermentation or used for the formation of more complex substances, i.e., esters. Moreover, in the case of *S. bayanus*, weight loss during fermentation process was strictly dependent on apple cultivar used for musts production, which could be related to the fact that sugar profile varies between tested cultivars (Table 2). It also seems that nitrogen compounds had a significant impact on the fermentation rate because their level was differed significantly among musts tested after fermentation (Table 1).

Malic acid is the major organic acid in apples. The titratable acidity of all analysed apple musts ranged from 4.38 to 6.29 g/L (Table 1) and, in the case of the Elise and Rubin cultivars, it decreased after fermentation. Some yeast species are able to assimilate malic acid. *S. cerevisiae* strains show various ability to degrade malic acid during alcoholic fermentation (up to 3 g/L of malic acid) [17]. Obtained results suggest that the profile of organic acids in tested apples was significantly different because, e.g., in the case of musts fermented with *S. bayanus*, titratable acidity after fermentation increased in the Elise and Topaz musts, while it decreased in the Rubin must. This would suggest that Rubin initially contained some acids which could be fermented by that yeast strain [18]. However, since the impact of particular organic acids was not the main focus of the current study, we did not investigate this aspect further.

Fermented apple musts contained from 5.0 (Elise, spontaneous fermentation) to 6.9% vol. ethanol (Topaz, *S. cerevisiae*) and the real fermentation efficiency was between 80.0% (Rubin, *S. bayanus*) to 92.0% (Topaz, *S. cerevisiae*). Theoretical efficiency of fermentation is 88% [19] and, in general, the higher ethanol content, the greater fermentation efficiency. In the case of all musts fermented by *S. cerevisiae* strain, the fermentation efficiency and content of ethanol were highest. Ethanol Red yeast demonstrates higher fermentation efficiency and tolerance to higher ethanol content—above 18% vol. ethanol [20]. This also supports the statement above—that *S. bayanus* uses up carbon sources on the production of volatile compounds more intensely than *S. cerevisiae*. Yeasts slow down their metabolism because the content of ethanol decreases their viability [8].

### 2.2. Volatile Compounds

Based on the two-way analysis of variance, it can be concluded that microorganism used for fermentation had a greater impact on the content of volatile compounds rather than apple cultivar (Table 3).

Esters were the most diverse group of analysed volatile compounds, consisting of almost 60 compounds, which were divided into four groups (methyl and ethyl esters, acetates, benzoates, and other esters).

Methyl and ethyl esters were the largest group to which 26 different compounds were assigned. The metabolism of methyl esters of fatty acids by yeast is seemingly unknown; however, yeast-mediated transesterification, or alcoholysis, is a probable route of formation of ethyl esters from methyl esters [21]. Methyl valerate was the sole compound from the methyl ester group which presence was dependent on apple cultivar. Methyl valerate was present only in apple spirits obtained from the Topaz cultivar, regardless of the type of fermentation, and its highest concentration was observed in samples fermented by *S. bayanus.* Similar results were obtained in previous studies [13] in which methyl valerate was present only in brandies derived from the Topaz cultivar (among 10 other cultivars). Methyl anthranilate is the basic aroma compound of *Vitis labrusca* and some other fruits, including several apple cultivars [22]. It is formed in a reaction catalysed by methanol acyltransferase from anthraniloyl-CoA and methanol and it has characteristic orange-flower aroma [23,24]. This compound was present in brandies obtained from musts fermented with *S. cerevisiae* (Ethanol Red). Similar results were obtained in previous studies [13] in which methyl anthranilate was present in all samples fermented with Ethanol Red.

Ye et al. (2014) [25] observed that total ethyl esters production, particularly ethyl lactate and ethyl butanoate, were highest in ciders produced by *Wickerhamomyces anomalus*/*S. cerevisiae* co-inoculation compared to a sole culture of *S. cerevisiae*. Moreover, Garavaglia et al. (2015) [26] proved that a mixed culture of *Debaryomyces vanriji* and *S. cerevisiae* had a positive impact on the concentration of esters, especially ethyl lactate, diethyl succinate, and ethyl decanoate, which have characteristic fruity aromas. In our studies, ethyl lactate and ethyl butanoate were present in highest concentrations in samples fermented spontaneously. At a concentration exceeding 250 mg/L, ethyl lactate has a negative effect on the flavour of brandies—it introduces buttery and rancid butter aromas. However, low concentrations (below 154 mg/L) are favourable and stabilise aroma. Its presence in alcoholic beverages could be linked to a malolactic fermentation, which is considered as a symptom of spoilage [27]. Ethyl esters of middle–chain fatty acids are compounds of particular interest in fermented beverages, and in brandies, they provide a fruity and flowery aroma. Ethyl caproate (banana, green apple, and melon aroma) and ethyl caprylate—more pungent and less fragrant [27]—were present in all analysed spirits, and their concentrations varied depending on the type of fermentation. The first one is the most abundant of all middle-chain fatty acid esters and the highest content of this compound characterised samples fermented with *S. cerevisiae*. The highest concentration of ethyl caprylate was determined in samples fermented spontaneously and the lowest in brandies obtained from musts fermented with *S. bayanus.* Some of the yeast strains produce large quantities of these compounds, which contribute to the fermentation aroma of apple brandies [1].

The second analysed group of esters in apple brandies were acetates. The acetic esters of higher alcohols are produced during the condensation of acyl-CoA and higher alcohols are formed by alcohol transferases (AATase). *S. cerevisiae* has two AATases (Atf1p and Atf2p), and in *S. bayanus* cells, another AATase (Lg-Atf1p) is present. Atf1p is the most important ester synthase for the production of acetate esters, e.g., isoamyl acetate, phenylethyl acetate, and, of C3 to C8, acetate esters [28]. For this reason, isoamyl acetate was detected in the highest concentration in brandies obtained from musts fermented by *S. bayanus*; nevertheless, its amount was also high in other samples (from 1.5 to 55.9 mg/L 100% vol. alcohol). Patelski et al. (2014) [29] also proved that plum brandies obtained from musts fermented by *S. bayanus* had higher amount of isoamyl acetate (8.00 mg/L 100% vol. alcohol), compared to spontaneous fermentation (3.01 mg/L 100% vol. alcohol). Similarly, 2-phenylethyl acetate was found in the highest concentration in spirits obtained from musts fermented by *S. bayanus* (Table 3). This compound is an important volatile in distillates with a rose and honey scent and a raspberry-like taste [30]. It can be concluded that *S. bayanus* is a good acetate esters producer. The exception was ethyl acetate, which was present in the highest concentration in samples fermented spontaneously. Satora and Tuszyński (2015) [31] claimed that non-*Saccharomyces* yeasts such as *Candida pulcherrima*, which triggered fermentation of apple wine, produce a considerable amount of ethyl acetate (200 mg/L), whereas a significantly lower amount of this compound (only 2 mg/L) is produced by *Saccharomyces* strains. Patelski et al. (2014) [29] presented a significantly higher concentration of ethyl acetate in plum brandies obtained from musts fermented spontaneously, compared to samples fermented by *S. bayanus.*

The third analysed group were benzoate esters which included ethyl benzoate, hexyl benzoate, and benzyl benzoate. The highest concentration of ethyl benzoate was observed in samples fermented spontaneously. According to Synos et al. (2015) [32], during spontaneous fermentation, more ethyl benzoate is produced in grape wines, compared to fermentation with *S. cerevisiae*.

GC–MS method enabled the detection of 23 other esters (Table 3). Butyl crotonate, isoamyl lactate, and phenethyl 2-methylbutyrate were detected only in samples fermented spontaneously. Ethyl (*Z*)-4-decenoate was present in spirits obtained from Topaz.

The second most abundant group of volatiles in apple brandies was alcohols. Methanol is formed during demethoxylation of esterified methoxyl groups in pectin [27]. The highest content of methanol was detected in samples fermented spontaneously, yet it never exceeded the maximum acceptable methanol content in apple spirits (12 g/L 100% vol. alcohol) [33]. Increased methanol content in brandies obtained from samples fermented spontaneously might be associated with high esterase activity, especially of microorganisms that initiate the fermentation.

Amyl alcohols were detected in higher concentrations in samples obtained from musts fermented spontaneously and in the lowest in samples fermented by *S. bayanus*. Presence of propanol, hexanol, heptanol, 1-octanol, and 1-nonanol resulted from the reduction of aldehydes to alcohols during fermentation [34]. These compounds were present in the highest concentration in brandies obtained from must fermented spontaneously, however propanol was also present in samples fermented with *S. bayanus.* It is claimed that *S. bayanus* yeast synthesises a high amount of propanol and it can produce even 50% more of this compound than *S. cerevisiae* [12]. In the analysed apple spirits, the concentration of hexanol varied from 6.5 to 31.7 mg/L 100% vol. alcohol. This observation was confirmed by Patelski et al. (2014) [29], who showed higher content of hexanol in plum brandies obtained from musts fermented spontaneously, compared to *S. bayanus*. Hexanol in a concentration above 100 mg/L has a negative effect on the flavour of brandies, as it produces liquorice and grassy aromas [27].

The next group of compounds in analysed apple brandies was aldehydes and ketones. The dominant compound was benzaldehyde (bitter almond, marzipan, and cherry aroma) [2,9], a high concentration of which was detected in samples fermented by *S. cerevisiae* and *S. bayanus*. This substance was present in a low concentration or absent in the samples fermented spontaneously. Some yeasts are able to utilise benzaldehyde in the presence of glucose and transform it into benzyl alcohol, benzoic acid, or other compounds [35]. Unlike benzaldehyde, the highest contents of acetaldehyde and furfural were detected in samples fermented spontaneously. There are large differences in acetaldehydes obtained by yeasts from pyruvate through the glycolytic pathway with concentrations ranging from 0.5 to 700 mg of acetaldehyde per liter [36]. Fermentation with *S. bayanus* is known to produce much more acetaldehyde in obtain wines than fermentation with *S. cerevisiae* [37]. Synos et al. (2015) [32] showed higher concentration of furfural in wine obtained after fermentation with *S. bayanus* than with *S. cerevisiae* or spontaneous fermentation. In our study, the lowest concentration of this compound was detected in samples fermented by *S. cerevisiae*. This compound normally occurs in fruit distillates and can be used as an indicator of distillate adulteration [27].

The final group relevant to the aroma of the analysed apple brandies are terpenes. α-phellandrene, (*E*)-β-famesene, (*Z*,*E*)-α-farnesene, α-farnesene, and farnesol were found in the highest concentrations in spirits obtained from musts fermented spontaneously. These results support other reports that linked non-*Saccharomyces* yeasts with higher β-glucosidase activities than *S. cerevisiae* yeasts and, thus, enriching the aroma of alcoholic beverages through releasing terpenoids [38]. However, *Saccharomyces cerevisiae* yeast endogenously synthesises precursors of most terpenoids, including GPP (geranyldiphosphate), FPP (farnesyldiphosphate), GGPP (geranylgeranyldiphosphate), and squalene [39]. Higher concentration of certain terpenes such as linalool oxide, linalool, guaiacol, citral, and β-ionone in samples fermented with *S. cerevisiae* might be linked with that pathway.

The presence of eugenol may be related to the presence of methyleugenol, which was detected in all analysed samples, except for spirits obtained from the Topaz cultivar fermented by *S. cerevisiae*. In turn, these samples showed the highest content of eugenol (Table 3). Methyleugenol is produced by the methylation of eugenol, hence, their occurrence may be codependent [24].

The highest amounts of β-citronellol in apple brandies obtained from musts fermented with *S. cerevisiae* might be connected with its formation by this strain of yeast. Carrau et al. (2017) [40] revealed that the presence of β-citronellol in alcoholic beverages could depend on the hydrolysis of glycosides with bound citronellol or transformation from geraniol and nerol carried out by *S. cerevisiae*. Very low content or absence of geraniol in analysed samples might correspond with its total biotransformation to citronellol by yeast cells. Moreover, according to Pardo et al. (2015) [41], geraniol can be metabolised by yeast enzymes to additional monoterpenes and esters, e.g., citronellol, linalool, nerol, citronellyl acetate, and geranyl acetate. Furthermore, linalool was present in higher concentration in samples fermented with *S. cerevisiae*. Camesasca et al. (2014) [42] showed that overexpression of COQ1 gene in *S. cerevisiae* significantly increased the formation of linalool and nerolidol. COQ1gene is connected with the synthesis of geranyl pyrophosphate and triggers nerolidol synthase activity under exponential growth conditions [40,42].

Apple cultivar had a greater impact on limonene concentration in analysed brandies than fermentation variant. This compound was absent in samples obtained from fermented Rubin musts. Similar results were obtained in previous studies [13], in which limonene was present only in samples produced from Elise and Topaz cultivars. Limonene is one of the most common compounds found in essential oils of aromatic plants, and this compound might have evolved (under acidic condition or in the presence of oxygen) into α-terpineol and carvone [13,43], which were detected in the highest concentrations in samples obtained from this cultivar.

### 2.3. Sensory Analysis

**Figure 2:**
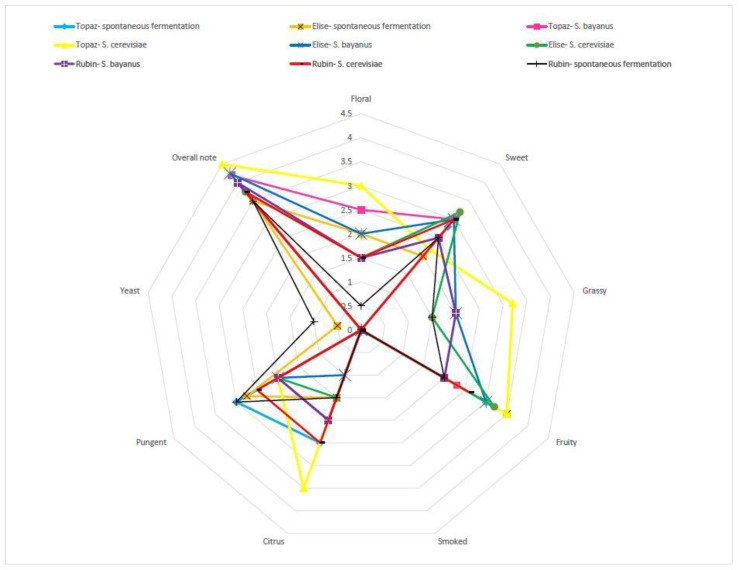
Characteristic aroma traits of apple spirits obtained from musts fermented by different microorganism, *n* = 5, STD < 5%.

All analysed samples were described as clear and obtained maximum scores for that parameter. The majority of samples received high scores for the parameter “overall note” (general acceptance of tested samples) from 3.5 to 4.5 in 5-point hedonistic scale (Figure 2). Higher scores for overall note (mean value 4.5) were characteristic for brandies obtained from the Topaz cultivar fermented by *S. cerevisiae*, and its aroma was described as floral, mildly sweet, grassy, fruity, pungent, and intensively citrus. This means that samples demonstrating those characteristics were more acceptable by panellists. Intensively citrus aroma could be the result of a higher concentration of citral (minimum 3 times higher concentration in tested samples, Table 3) and limonene in samples obtained from the Topaz must fermented by *S. cerevisiae*, compared with samples obtained from other fermentation types (Table 3). These two compounds are associated with orange, lemon, and citrus aromas, and its aroma threshold varies from 4 to 229 ppb [24]. The grassy aroma recognised in this sample (Topaz, *S. cerevisiae*) could be associated with the highest concentration of linalool and linalool oxide, which give earthy, floral, herbal, and lavender scents; the taste threshold value is 5 ppm [24,30], and in analysed samples, that value was exceeded, which means that it had significant impact on flavour. Floral and sweet aroma could be associated with the presence of geraniol (sweet, rose, and geranium aroma; taste threshold values at 10 ppm) and eugenol (cloves scent; detection threshold aroma 6 to 100 ppb) [24,30], which appeared in the highest concentrations in this sample (Topaz, *S. cerevisiae*).

Brandies produced from musts fermented with *S. bayanus* obtained the highest average scores for “overall note” (more than 4.0 pt) regardless of the apple cultivar used for the distillery industry. It means that this strain of yeast could be feasible for the distillery industry. That strain was shown to be the best acetate ester producer (Table 3) and those compounds are associated with a flower and fruit aroma which is considered acceptable. It finds confirmation in the Pearson test because floral aromas were strongly associated with distillates from all apple cultivars fermented with *S. bayanus* (Table S1, Supplementary Materials).

In turn, the lowest scores for “overall note” characterized the samples that were obtained from musts fermented spontaneously, which had the most intense pungent aroma, which may be caused by higher concentrations of methanol, acetaldehyde, and fusel alcohols in these samples. The Pearson test indicated positive correlations between some descriptors (floral, sweet, fruity, or citrus) and overall note. Strongest positive correlations for citrus aroma were noted for samples of Elise and Rubin fermented spontaneously and Rubin fermented with *S. bayanus*. Moreover, there were negative correlations between pungent and, in some cases, yeast descriptors and overall note (Table S1, Supplementary Materials).

## 3. Materials and Methods

### 3.1. Fermentation

Apple musts used for the fermentation were obtained from Elise, Rubin and Topaz cultivars harvested from orchards in Garlica Murowana (Kraków, Poland). Apples were washed, dried with paper towel, crushed manually, and pressed with a Zottel hydraulic press (35 L) (Zottel Trade d.o.o., Žalec, Slovenia), and musts were divided into 2 kg portions in 3 L glass flasks. Each fermentation variant was prepared in five replicates. Musts were supplemented with (NH_4_)_2_HPO_4_ (0.2 g/kg raw fruit) and fermented spontaneously or inoculated (0.3 g d.w./L of must) with Ethanol Red (*Saccharomyces cerevisiae*) yeast strain (Starowar, Warsaw, Poland) or cider yeast (*Saccharomyces bayanus*) (Young’s brew, Bilston, England). Yeasts were rehydrated according to the manufacturer’s instructions. Flasks were capped with a plug containing a fermentation tube filed with glycerol. Alcoholic fermentation was carried out for 30 days at 20 °C. The temperature of the room where flasks were stored was controlled daily. Weight loss associated with the liberation of carbon dioxide was measured daily. After fermentation samples for chemical analysis (sugar profile, glycerol concentration, total extract, sugar-free extract, titratable acidity, and free amino nitrogen) were collected and stored at −20 °C until analysis. The remaining volume of must was immediately distilled for further analysis.

### 3.2. Distillation

Fermented musts were distilled immediately after fermentation terminated. The distillation was stopped when ethanol concentration in the collected distillate was lower than 0.5% [44,45,46] (*w*/*v*). Final ethanol concentration was ranging between 10.8–20.1% (*v*/*v*) of ethanol.

Then, the distillate was distilled again using a glass column (40 cm) filled up to 60% with Raschig rings. During the second distillation, three fractions were collected: the heads (2% of the distillate, ethanol concentration 80% (*v*/*v*)), the heart fraction (83% of the distillate, ethanol concentration 65% (*v*/*v*), and the tails (15% of the distillate, ethanol concentration 20% (*v*/*v*)). In order to avoid the loss of volatiles, all fractions were kept at 4 °C to further analysis. In the current study, we only presented results for heart fraction.

### 3.3. Analysis of Oenological Parameters

After fermentation, the ethyl alcohol content, total extract content, sugar-free extract and titratable acidity were determined using officially approved methods [47]. Titratable acidity was determined with the potentiometric method and was calculated from the volume of 0.1 M NaOH used for the titration and expressed as g of malic acid per L. The fermentation efficiency [%] was calculated based on the relationship between sugar consumed and ethyl alcohol produced following the fermentation stoichiometry, where 0.511 g of ethyl alcohol is obtained from 1 g of reducing sugar and 0.538 g ethyl alcohol from 1 g of sucrose. Free amino nitrogen (FAN) was determined with the ninhydrin method. The absorbance of the samples was analysed at a wavelength λ = 575 nm [48].

### 3.4. Determination of Sugar Content by High-Performance Liquid Chromatography (HPLC)

Apple musts before and after fermentation were centrifuged (MPW-65R, MPW Med. Instruments, Warszawa, Poland) at 14,000× *g*/5 min and fresh musts were diluted with deionized water. Fermented musts were evaporated (Rotavapor R-220 SE, Buchi AG, Flawil, Switzerland) prior to analysis because the concentration of sugars in this type of samples is very low, and through evaporation, we managed to detect tested sugars above detection limits of the used method. Before injecting (10 μL), samples were filtered through syringe filters (0.45 μm pore density, Sartorius AG, Getinge, Germany). Sugar profile analysis was determined by HPLC method using a Shimadzu apparatus (Kyoto, Japan) NEXERA XR equipped with the refractometer detector RF-20A. The separation was conducted with an Asahipak NH2P-50, 4.6 × 250 mm Shodex column (Showa Denko America, Germany) thermostated at 30 °C. An aqueous solution of acetonitrile (70%) was the mobile phase and the isocratic elution program (0.8 mL/min) lasted 16 minutes. The standard curve was prepared for glucose, fructose, sucrose, and glycerol [13].

### 3.5. Volatile Compounds Analysis by GC–FID (Gas Chromatography–Flame Ionization Detector) and SPME–GC–MS (Solid Phase Microextraction–Gas Chromatography–Mass Spectrometry)

Selected volatile compounds were analysed using GC–FID as described by Satora et al. (2008) [20]. The analysis was carried out on the Hewlett Packard 5890 Series II chromatograph system (Agilent Technologies, Santa Clara, CA, USA). The separation was conducted with an HP-INNOVAX capillary column (crosslinked polyethylene glycol stationary phase; 30 m × 0.53 mm ID with 1.0 μm film thickness). Temperature of detector and injector was 250 °C and the column was heated using the following program: 35 °C for five minutes at an increment of 5 °C/min to 110 °C, then 40 °C/min to 220 °C and maintaining a constant temperature for three minutes. The carrier gas was helium at a 20.0 mL/min flow. Hydrogen flow speed was 33.0 mL/min, and that of air was 400 mL/min. The qualitative and quantitative identification of volatile compounds and internal standard (anethole, ethyl nonanoate, and 4-methylo-2-pentanol) was based on the comparison of retention times and peak surface area read from sample and standard chromatograms. Concentrations of volatile components were recalculated based on 100% (*v*/*v*) ethanol and were expressed as mg/L. All tests were carried out three times.

In the SPME–GC–MS method, 2 mL of saturated saline with an internal standard solution (5 mg of 4-methyl-2-pentanol/L and 0.05 mg of ethyl nonanoate/L, Sigma-Aldrich, Saint Louis, MO, USA) and 0.05 mL of spirit were added into a 10 mL vial. The SPME device (Supelco Inc., Bellefonte, PA, USA) coated with polydimethylsiloxane (100 μm) fiber was first conditioned by inserting it into the GC injector port at 250 °C for 1 h. For sampling, the fiber was inserted into the headspace under stirring (300 rpm) for 30 min at 60 °C. Subsequently, the SPME device was introduced in the injector port of the Agilent Technologies 7890B chromatograph system (Agilent Technologies) equipped with LECO Pegasus HT, High Throughput TOFMS (time-of-flight mass spectrometry), and was kept in the inlet for 3 min. The SPME process was automated using the GERSTEL MultiPurpose Sampler (MPS).

Separation was conducted with a Rtx-1ms capillary column (Crossbond 100% dimethyl polysiloxane, 30 m × 0.53 mm × 0.5 μm). The detector temperature was 250 °C, and the column was heated using the following program: 40 °C for three minutes at an increment of 8 °C/min to 230 °C, then maintaining a constant temperature for 9 min. The carrier gas was helium at a 1.0 mL/min constant flow. EIMS electron energy 70 eV; ion source temperature and connection parts: 250 °C. Analyte transfer was performed in splitless mode; the MSD (mass spectrometer detector) was set to scan mode from *m*/*z* = 40 to *m*/*z* = 400.

Volatiles were identified using mass spectral libraries and linear retention indices, calculated from a series of n-alkanes from C6 to C30. The amount of components was determined semi-quantitatively by measuring the relative peak area of each identified compound, according to the NIST database, in relation to that of the internal standard (ethyl nonanoate for esters, 4-methyl-2-pentanol for other components). This semi-quantification approach was already performed in many previous scientific studies [49,50,51].

### 3.6. Sensory Analysis

Sensory analysis of apple brandies was based on eight aroma descriptors (fruity, sweet, grassy, floral, smoked, citrus, pungent, and yeast) rated on a five-point hedonistic scale. For overall note, 0 meant least acceptable and 5 most acceptable. For aroma descriptors, 0 was used for the least intensity of aroma and 5 was used for the highest intensity of a particular aroma. We applied quantitative descriptive analysis (QDA). The overall note in our paper defines the general acceptance of tested samples. Panellists were selected among scientific staff working in the Faculty of Food Technology and Human Nutrition who previously graduated from that faculty and obtained extensive course of sensory analysis as a part of their curriculum. Sensory assessment was determined using a set of standards provided to panellists prior to analysis [13,52]. First, panellists received standards of various aromas to determine whether they were able to recognize each of them. Then, they received the same standards, but at various concentrations. Only those who passed those two stages were selected as panellists. Apple brandies (diluted to 40% vol. EtOH) were subjected to sensory assessment by the panel comprising of 10 panellists (5 females and 5 males in age ranging from 20 to 35). Samples were coded and provided to panellists in randomized order in 50 mL laboratory beakers. Results were subjected to one-way analysis of variance (ANOVA) and then the Pearson test was carried out for each descriptor (Table S1, Supplementary Materials).

### 3.7. Statistical Analysis

All experiments were conducted in at least five physical replicates and each analysis was carried out for each replicate. Results were presented as arithmetic means ± standard deviation. Statistical analysis was performed in the R 3.6.1 (Viena, Austria) program. We applied the Shapiro–Wilk test to assess the normality of data distribution. Then, we carried out analysis of multivariate analysis of variance (MANOVA) with the post hoc Tukey test.

## 4. Conclusions

Our research confirmed the hypothesis that various types of fermentation significantly influence chemical composition of fermented musts, as well as the volatile profile and sensory characteristics of obtained spirits, and that *S. bayanus* is an appropriate strain of yeast for brandies production. In musts fermented spontaneously, the adaptation period of microorganisms to fermentation conditions was the longest and fermentation was slower in comparison to other tested variants. Samples fermented by *S. cerevisiae* demonstrated the highest fermentation efficiency and ethanol content. In our study, the best acetate ester producer was *S. bayanus*. The exception was ethyl acetate, which was present in the highest concentration in samples fermented spontaneously. Highest concentrations of acetaldehyde, methanol, and a majority of fusel alcohols were in samples obtained by spontaneous fermentation. Terpenes were found in the highest concentrations in spirits obtained from musts fermented spontaneously. These results support other reports that linked non-*Saccharomyces* yeasts with higher β-glucosidase activities, enhancing the aromas of alcoholic beverages through releasing aroma compounds from glycosidic forms. Apple brandies obtained from the Topaz cultivar fermented by *S. cerevisiae* demonstrated the most diverse profile of volatile compounds and the best aroma, described as floral, mildly sweet, grassy, fruity, alcoholic, and intensively citrus. Moreover, their oenological parameters that are the most important in the production of alcoholic beverages were the most favourable. Sensory results justify application of *S. bayanus* for the production of apple brandies because they improve the quality of alcoholic beverages. Brandies obtained from musts fermented by *S. bayanus* received the highest average score for the “overall note” parameter. In overall, apple brandies obtained after spontaneous fermentation obtained lower results in sensory analysis. It also must be considered that in many cases, products, including beverages, obtained in this type of processes might pose potential risks to human health due to the presence of some toxic substances (e.g., ethyl carbamate) or some pathogens (e.g., *Escherichia coli* O157:H7) [53,54].

However, further studies should be carried out to evaluate if the obtained profiles of volatile compounds of apple brandies might be associated with the presence of particular microorganisms rather than the applied apple cultivar.

## Figures and Tables

**Table 1:** Chemical composition of fresh and fermented apple musts obtained from Topaz, Rubin and Elise cultivars fermented with different type of yeasts.

**Before Fermentation Process**							
	**Parameters**	**Total Extract**	**Sugar-Free Extract**	**Titratable Acidity**	**Free Amino Nitrogen (FAN)**		
**Apple Cultivars**		**[g/L]**			**[mg/L]**		
**ELISE**		97.0c ± 1.0	13.4a ± 1.02	4.38b ± 0.07	51.5c ± 0.1		
**RUBIN**		108.0b ± 1.0	9.9c ± 1.21	6.29a ± 0.62	54.4b ± 0.1		
**TOPAZ**		124.0a ± 1.0	12.9ab ± 1.01	5.00b ± 0.04	59.5a ± 0.2		
**Significance**		***	***	**	***		
**After Fermentation Process**							
	**Parameters**	**Total Extract**	**Sugar-Free Extract**	**Titratable Acidity**	**Free Amino Nitrogen (FAN)**	**Ethanol Content**	**Fermentation Efficiency**
**Type of Fermentation**		**[g/L]**			**[mg/L]**	**[% vol.]**	**[%]**
**ELISE**	**SF**	12.0Cc ± 1.0	10.4Ce ± 0.9	3.83Ccd ± 0.26	27.9Aa ± 4.4	5.0Cde ± 0.1	89.3ABb ± 0.2
	**SC**	19.0Cc ± 2.0	17.4BCcd ± 0.9	3.93Ccd ± 0.86	22.0Bb ± 2.4	5.1Cf ± 0.1	90.9Aa ± 0.4
	**SB**	17.0Cc ± 3.0	15.9Cd ± 0.9	4.69BCbc ± 0.68	23.4Bab ± 4.7	5.1Cf ± 0.1	90.5Aa ± 0.2
**RUBIN**	**SF**	21.0Bc ± 6.0	19.6Bc ± 0.9	3.46Ccd ± 0.29	22.4Bb ± 1.1	5.5BCd ± 0.2	83.2Bc ± 1.5
	**SC**	25.0ABab ± 3.0	22.9Aa ± 0.6	4.12Cbcd ± 0.62	28.3Aa ± 5.1	5.9Bc ± 0.2	88.5ABb ± 1.1
	**SB**	23.0Bbc ± 2.0	19.9Bbc ± 1.0	2.71Dd ± 0.16	21.8Bb ± 4.9	5.4BCe ± 0.1	80.0Bd ± 1.1
**TOPAZ**	**SF**	28.0Aa ± 1.0	24.4Aa ± 0.1	5.47Bb ± 0.45	29.4Aa ± 1.4	6.7Ab ± 0.1	89.3ABb ± 0.8
	**SC**	27.0Aa ± 2.0	24.4Aa ± 0.1	7.41Aa ± 0.62	22.0Bb ± 5.7	6.9Aa ± 0.1	92.0Aa ± 1.3
	**SB**	26.0Aab ± 2.0	22.5Aab ± 0.1	7.36Aa ± 0.52	24.0Bab ± 7.3	6.8Ab ± 0.1	90.7ABa ± 1.8
**Significance**	**Cultivars**	***	*	*	***	**	***
	**Fermentation variant**	**	***	**	***	***	***

Same letters next to mean values within columns indicate the lack of statistically significant differences at *p* < 0.05; *n* = 5; *ns*—not significant; 0.001 ‘***’; 0.01 ‘**’; 0.05 ‘*’. Abbreviations: SF—spontaneous fermentation, SC—fermentation with *Saccharomyces cerevisiae* (Ethanol Red), SB—fermentation with *Saccharomyces bayanus* (cider yeast). Capital letters were used to mark differences for apple cultivar, while lowercase letters were used for fermentation variant.

**Table 2:** Sugars composition of fresh and fermented musts obtained from Topaz, Rubin, and Elise cultivars fermented with different type of yeasts.

**Before Fermentation Process**						
		**Glycerol**	**Fructose**	**Glucose**	**Sucrose**	**Total Sugars**
		**[g/L]**				
**ELISE**		nd	53.12c ± 1.26	13.13c ± 0.11	17.39b ± 0.11	83.64c ± 2.47
**RUBIN**		nd	69.33a ± 0.98	18.43b ± 2.00	10.38c ± 0.57	98.14b ± 3.55
**TOPAZ**		nd	61.33b ± 1.55	23.98a ± 0.90	25.72a ± 0.92	111.03a ± 3.37
**Significance**		ns	***	***	***	***
**After Fermentation Processes**						
		**Glycerol**	**Frutose**	**Glucose**	**Sucrose**	**Total** **Sugars**
		**[g/L]**				
**ELISE**	**SF**	4.35Aab ± 0.81	1.33Ba ± 1.18	0.17Aa ± 0.12	0.09Ab ± 0.02	5.94BCa ± 1.32
	**SC**	4.87Aab ± 1.27	1.10Ba ± 0.13	0.26Aa ± 0.08	0.24Aab ± 0.05	6.47Ba ± 0.26
	**SB**	4.30Aab ± 0.70	0.87Ba ± 0.23	0.08Aa ± 0.03	0.10Ab ± 0.05	5.35Ca ± 0.31
**RUBIN**	**SF**	3.63Ab ± 0.61	1.11Ba ± 0.25	0.10Aa ± 0.08	0.17Ab ± 0.04	5.01Ca ± 0.37
	**SC**	5.21Aab ± 0.86	1.20Ba ± 0.18	0.03Aa ± 0.04	0.18Ab ± 0.07	6.62Ba ± 0.29
	**SB**	5.83Aa ± 0.78	1.52Ba ± 0.24	0.20Aa ± 0.15	0.36Aab ± 0.13	7.91Aa ± 0.52
**TOPAZ**	**SF**	5.10Aab ± 0.34	3.24Aa ± 0.52	0.20Aa ± 0.13	0.11Ab ± 0.03	8.65Aa ± 0.68
	**SC**	5.79Aa ± 0.57	2.25ABa ± 0.39	0.13Aa ± 0.01	0.13Ab ± 0.06	8.30Aa ± 0.46
	**SB**	4.71Aab ± 0.44	2.21ABa ± 0.40	0.68Aa ± 0.29	0.58Aa ± 0.35	8.18Aa ± 1.04
**Significance**	**Cultivars**	ns	***	ns	ns	0.05
	**Fermentation variant**	**	ns	ns	*	ns

Same letters next to mean values within columns indicate the lack of statistically significant differences at *p* < 0.05; *n* = 5; *ns*—not significant; 0.001 ‘***’; 0.01 ‘**’; 0.05 ‘*’. Abbreviations: SF—spontaneous fermentation, SC—fermentation with *Saccharomyces cerevisiae* (Ethanol Red), SB—fermentation with *Saccharomyces bayanus* (cider yeast). Capital letters were used to mark differences for apple cultivar, while lowercase letters were used for fermentation variant.

**Table 3:** Aroma composition of apple spirits obtained from musts fermented with different type of yeast [mg/L 100% vol. alcohol].

	LRI ^1^	ELISE			RUBIN			TOPAZ			Significance	Method
		SF	SC	SB	SF	SC	SB	SF	SC	SB		
**Methyl and ethyl esters**												
*n*-Ethyl propanoate	678	39.3a	67.1a	14.0a	31.1a	12.1a	41.2a	90.2a	71.5a	16.3a	ns	GC–MS
Ethyl butanoate	789	284.2a	162.5b	117.4bc	203.9ab	140.2bc	103.6c	328.1a	183.4b	31.3c	***	GC–MS
Ethyl lactate	797	230.7a	0.0c	16.8b	192.6a	58.7b	0.0c	131.0a	0.0c	0.0c	***	GC–MS
Ethyl (*Z*)-2-butenoate	822	30.1b	17.7b	0.0b	74.2ab	0.0b	0.0b	181.7a	0.0b	0.0b	**	GC–MS
Methyl valerate	823	0.0	0.0	0.0	0.0	0.0	0.0	25.5bc	58.8b	127.3a	***	GC–FID, GC–MS
Ethyl 2-methylbutanoate	841	32.6b	18.0b	0.0b	6.3b	0.0b	9.3b	183.8a	3.0b	1.2b	**	GC–MS
Ethyl 3-methylbutanoate	843	0.0b	3.4ab	0.0b	0.0b	0.0b	11.3a	0.0b	1.3b	0.9b	**	GC–MS
Methyl caproate	907	58.5b	75.8a	0.0d	0.0d	0.0d	19.7bc	34.1b	4.5d	0.0d	***	GC–MS
Ethyl caproate	986	24.1cd	30.9bc	26.1cd	17.7d	21.7cd	20.1cd	41.2ab	51.5a	31.0bc	***	GC–FID, GC–MS
Methyl caprylate	1108	469.7a	676.3a	22.4c	13.9c	6.5c	123.3b	131.1b	18.6c	10.2c	***	GC–MS
Diethyl succinate	1149	0.54c	1.02bc	2.71a	0.35c	2.04ab	0.76c	0.75c	0.47c	0.69c	**	GC–FID, GC–MS
Ethyl caprylate	1180	10.8a	6.1b	5.1b	8.4a	6.7ab	4.8bc	8.7ab	1.4c	0.5c	***	GC–FID, GC–MS
Ethyl phenylacetate	1210	86.9a	98.1a	9.4a	110.9a	6.9a	18.7a	87.6a	69.2a	51.6a	ns	GC–MS
Methyl decanoate	1330	3.4a	6.4a	0.8a	0.2a	0.2a	0.9a	0.9a	0.3a	0.1a	ns	GC–MS
Methyl anthranilate	1339	0.0d	91.2a	53.9b	0.0d	73.9ab	0.0d	23.0c	60.8b	0.0d	***	GC–FID, GC–MS
Ethyl (*Z*)-4-decenoate	1357	0.0b	0.0b	0.0b	0.0b	0.0b	0.0b	0.017a	0.019a	0.037a	***	GC–MS
Methyl laurate	1507	2.26a	1.83a	0.54a	0.21a	0.16a	0.36a	0.51a	0.22a	0.17a	ns	GC–MS
Methyl dihydrojasmonate	1649	0.07a	0.01a	0.01a	0.02a	0.01a	0.01a	0.02a	0.01a	0.02a	ns	GC–MS
Methyl tetradecanoate	1707	0.21a	0.18a	0.12a	0.10a	0.03a	0.04a	0.13a	0.04a	0.04a	ns	GC–MS
Ethyl pentadecanoate	1880	0.07ab	0.05a	0.33a	0.03b	0.05b	0.08ab	0.12ab	0.05b	0.09ab	*	GC–MS
Methyl palmitate	1927	0.29a	0.23a	0.41a	0.1a	0.17a	0.15a	0.26a	0.17a	0.19a	ns	GC–MS
Ethyl *E*-11-hexadecenoate	1974	0.04a	0.38a	0.45a	0.02a	0.13a	0.09a	0.04a	0.38a	0.24a	ns	GC–MS
Ethyl palmitate	1990	7.8ab	6.1ab	18.8a	3.4b	4.9b	5.4ab	8.2ab	6.4ab	8.6ab	*	GC–MS
Methyl linoleate	2092	0.48a	0.29a	0.36a	0.35a	0.49a	0.31a	0.39a	0.24a	0.33a	ns	GC–MS
Ethyl elaidate	2171	0.023a	0.017a	0.024a	0.011a	0.014a	0.013a	0.031a	0.030a	0.029a	ns	GC–MS
Ethyl stearate	2189	0.047a	0.017a	0.052a	0.027a	0.031a	0.025a	0.051a	0.029a	0.052a	ns	GC–MS
**Acetates esters**												
Ethyl acetate	614	138.1a	130.2a	126.4ab	129.2ab	100.2d	103.5d	117.3bc	97.5d	93.1d	***	GC–FID, GC–MS
Isopropyl acetate	662	1.81b	0.22b	0.13b	13.41a	0.33b	0.26b	0.39b	0.17b	0.14b	**	GC–FID, GC–MS
Isobutyl acetate	763	24.4ab	65.5a	0.0b	4.5ab	9.3ab	5.2ab	4.7ab	7.2ab	1.4ab	*	GC–MS
Butyl acetate	799	0.0a	9.9a	0.0a	0.0a	0.0a	10.1a	0.0a	25.6a	6.8a	ns	GC–MS
Isoamyl acetate	876	12.7c	5.1c	21.8abc	14.5bc	1.5c	25.6abc	49.2ab	54.3a	55.9a	**	GC–FID, GC–MS
2-Methyl-1-butyl acetate	879	12.1ab	8.2a	0.0b	0.0b	2.9b	11.14ab	7.28ab	5.24b	0.0b	*	GC–MS
Hexyl acetate	1006	35.9c	108.9b	34.3c	0.0d	89.8bc	146.83ab	3.51d	230.78a	48.07c	**	GC–MS
Octyl acetate	1196	0.0b	26.6a	4.0b	0.0b	6.6ab	29.6ab	0.0b	14.5ab	5.9b	*	GC–MS
2-phenylethyl acetate	1228	100.3b	104.3a	114.1a	100.8b	101.1b	107.5ab	98.2b	101.3b	107.4a	***	GC–FID, GC–MS
**Benzoates**												
Ethyl benzoate	1142	34.5ab	25.9ab	0.0b	5.5b	0.0b	0.0b	91.6a	16.9ab	7.1b	**	GC–MS
Hexyl benzoate	1555	0.11a	0.15a	0.04a	0.02a	0.01a	0.01a	0.12a	0.06a	0.05a	ns	GC–MS
Benzyl benzoate	1750	0.021a	0.014a	0.031a	0.011a	0.002a	0.003a	0.026a	0.010a	0.017a	ns	GC–MS
**Other esters**												
Buthyl crotonate	1023	26.5a	0.0b	0.0b	16.5a	0.0b	0.0b	39.1a	0.0b	0.0b	***	GC–MS
Isoamyl lactate	1064	99.4a	0.0b	0.0b	37.2ab	0.0b	0.0b	26.3b	0.0b	0.0b	*	GC–MS
Hexyl butanoate	1174	0.0b	17.9a	0.9b	0.9b	0.5b	9.4ab	2.4b	3.1ab	8.6ab	**	GC–MS
Hexyl 2-methylbutanoate	1222	392.2a	218.9a	19.8c	9.1c	8.1c	16.3c	102.8b	7.6c	11.5c	*	GC–MS
Isopentyl hexanoate	1238	23.6a	26.7a	15.4a	4.1a	10.5a	29.1a	17.5a	23.7a	9.2a	ns	GC–MS
Isobutyl caprylate	1341	0.09ab	0.43a	0.02b	0.02b	0.02b	0.03ab	0.04ab	0.01b	0.01b	*	GC–MS
Hexyl hexanoate	1372	0.01b	0.31a	0.01b	0.0b	0.01b	0.01b	0.01b	0.03ab	0.04ab	*	GC–MS
β-Phenylethyl butyrate	1411	0.0b	0.04b	0.0b	0.17a	0.01b	0.0b	0.05b	0.03b	0.01b	***	GC–MS
Isopentyl octanoate	1445	0.39a	0.81a	0.47a	0.22a	0.39a	0.60a	1.19a	0.78a	0.24a	ns	GC–MS
2-Methylbutyl octanoate	1449	0.43a	0.75a	0.01a	0.02a	0.04a	0.07a	0.21a	0.08a	0.03a	ns	GC–MS
Phenethyl 2-methylbutyrate	1466	0.09ab	0.05ab	0.01b	0.03ab	0.01b	0.01b	0.16a	0.01b	0.01b	**	GC–MS
Propyl decanoate	1472	0.03ab	0.05a	0.03ab	0.01b	0.01b	0.03ab	0.01b	0.02ab	0.01b	**	GC–MS
Isobutyl decanoate	1546	0.76a	0.63a	0.07a	0.06a	0.04a	0.05a	0.18a	0.07a	0.02a	ns	GC–MS
Hexyl octanoate	1565	0.06a	0.07a	0.01a	0.02a	0.01a	0.01a	0.04a	0.02a	0.01a	ns	GC–MS
2-Phenylethyl hexanoate	1611	0.16bc	0.31bc	0.04c	0.22bc	0.11c	0.05c	0.47ab	0.71a	0.15bc	***	GC–MS
Isopropyl dodecanoate	1617	0.0a	0.0a	0.0a	0.01a	0.0a	0.0a	0.02a	0.01a	0.01a	ns	GC–MS
Isoamyl decanoate	1641	0.74a	0.91a	0.18a	0.78a	0.77a	0.85a	0.80a	2.12a	0.74a	ns	GC–MS
Methyl 8-(2-furyl) octanoate	1675	0.01b	0.15a	0.03b	0.02b	0.04b	0.01b	0.03b	0.03b	0.03b	**	GC–MS
Isobutyl laurate	1753	0.07a	0.05a	0.03a	0.02a	0.02a	0.01a	0.04a	0.01a	0.01a	ns	GC–MS
Hexyl decanoate	1784	0.07a	0.05a	0.03a	0.01a	0.02a	0.02a	0.05a	0.03a	0.03a	ns	GC–MS
Ethyl tetradecanoate	1790	7.7a	4.1a	6.9a	3.3a	1.7a	2.2a	5.5a	1.6a	2.6a	ns	GC–MS
Phenethyl octanoate	1820	0.49a	0.48a	0.13a	0.52a	0.26a	0.44a	0.61a	0.59a	0.28a	ns	GC–MS
Isoamyl laureate	1844	0.04a	0.02a	0.03a	0.02a	0.22a	0.03a	0.02a	0.15a	0.03a	ns	GC–MS
**Alcohols**												
Methanol	361	4956a	4773ab	3510bc	3716b	3492c	3676bc	4005ab	3027c	3451c	***	GC–FID, GC–MS
Propanol	568	2.5a	1.3b	2.2a	2.2a	1.4b	1.9a	1.8a	1.4b	1.7ab	*	GC–FID, GC–MS
Isobutanol	617	344a	307a	196a	199a	252a	230a	295a	304a	184a	*	GC–FID
Butanol	658	154a	54de	223b	47e	46e	40e	108cd	123c	95cde	*	GC–FID
Amyl alcohols	723	2042bc	1773a	1454ef	1973abc	1910abc	1669de	2109a	1632def	1444f	***	GC–FID, GC–MS
3-Methyl-1-pentanol	825	28.9c	29.5c	9.3c	11.9c	31.2c	9.9c	82.7b	125.4a	14.4c	***	GC–MS
3-Hexen-1-ol	845	1.30	3.4b	0.0b	28.7a	25.2a	61.7a	2.7b	3.1b	10.3a	ns	GC–MS
Hexanol	865	31.7a	16.4bc	22.7ab	15.4bc	11.17bc	6.5c	23.8ab	12.2bc	7.3c	***	GC–FID, GC–MS
Heptanol	954	19.1ab	9.4ab	1.2b	11.6ab	1.0b	6.3ab	36.1a	19.3ab	20.1ab	*	GC–MS
6-Methyl-5-hepten-2-ol	974	0.0a	2.2a	0.0a	8.7a	3.2a	3.0a	4.7a	1.4a	0.0a	*	GC–MS
3-Ethyl-4-methylpentanol	1011	56.8a	16.1ab	17.4ab	0.0b	0.0b	0.0b	0.0b	0.0b	0.0b	**	GC–MS
1-Octanol	1070	85.1ab	54.4ab	10.3ab	24.9ab	0.0b	18.9ab	104.5a	36.5ab	35.5ab	*	GC–MS
Phenylethanol	1084	213.8a	333.7a	114.3a	304.4a	487.7a	914.4a	476.7a	614.6a	343.2a	ns	GC–MS
1-Nonanol	1156	17.5ab	2.8b	0.0b	40.0a	7.4ab	23.5ab	19.2ab	2.4b	10.2ab	*	GC–MS
1-Decanol	1272	24.8a	23.0a	19.3a	24.4a	60.6a	16.1a	18.9a	15.7a	16.2a	ns	GC–MS
1-Tetradecanol	1664	0.13ab	0.05ab	0.03ab	0.02ab	0.04ab	0.02b	0.22a	0.02b	0.04ab	**	GC–MS
**Aldehydes and ketones**												
Acetaldehyde	412	201.1a	164.2b	154.3b	194.2a	168.4b	176.4ab	187.2a	143.2b	155.6b	***	GC–FID
Furfural	804	25.1a	20.8a	23.3a	53.5a	5.6b	28.4a	31.1a	0.0b	7.6b	***	GC–MS
Benzaldehyde	925	0.0c	879.5a	536.9ab	7.8c	361.3abc	792.4ab	11.0c	65.6c	251.3bc	***	GC–MS
Isovaleraldehyde	953	67.7a	80.0a	8.5b	3.5b	2.3b	18.2ab	57.0a	3.8b	0.0b	*	GC–MS
6-Methyl-5-hepten-2-one	967	67.6ab	48.2ab	7.7b	18.7ab	12.1b	23.9ab	15.5b	103.7a	68.9ab	*	GC–MS
2-Furaldehyde diethyl acetal	1078	3.4b	8.5ab	5.3ab	5.6ab	2.8b	0.8b	12.9a	4.7b	5.7ab	**	GC–MS
Nonanal	1083	143.2a	109.1a	11.5b	14.7b	6.4b	52.4ab	22.1b	10.1b	11.5b	**	GC–MS
Decanal	1182	2.8a	1.2a	2.5a	1.3a	0.9a	0.0a	0.0a	0.0a	1.8a	ns	GC–MS
**Terpenoids**												
α-Phellandrene	1003	50.9a	37.1a	0.0b	13.3ab	0.4b	3.4b	34.8a	8.6b	0.0b	**	GC–MS
*p*-Cymene	1014	34.4a	25.6a	0.0b	0.8b	0.5b	7.8b	11.2ab	2.6b	0.0b	**	GC–MS
Limonene	1020	0.21ab	0.10bc	0.25ab	0.00d	0.00d	0.00d	0.22ab	0.26a	0.09bc	**	GC–FID, GC–MS
Linalool oxide	1078	1.11a	1.19a	1.12a	0.28cd	0.46bc	0.39bc	0.59b	0.69b	0.63b	***	GC–FID, GC–MS
Linalool	1094	1.53b	2.39a	1.59b	0.20d	0.29cd	0.13d	0.61cd	0.82c	0.66cd	***	GC–FID, GC–MS
Guaiacol	1095	0.56b	1.08a	0.56b	0.42b	0.58b	0.48b	0.62b	0.71ab	0.46b	***	GC–FID, GC–MS
α-Terpineol	1171	2.2a	3.9a	0.8ab	5.3a	4.2a	4.6a	0.0b	0.0b	5.3a	**	GC–MS
(+)-terpinen-4-ol	1181	0.11b	0.12b	0.24ab	0.29ab	0.35a	0.13b	0.37a	0.51a	0.57a	**	GC–FID, GC–MS
(−)-β-citronellol	1229	0.04c	0.21b	0.06c	0.32ab	0.47a	0.42a	0.04c	0.49a	0.08c	***	GC–FID, GC–MS
Citral	1240	0.02e	0.39bcd	0.00e	0.02d	0.07cd	0.04d	0.42c	1.27a	0.50bc	***	GC–FID, GC–MS
Geraniol	1258	0.00c	0.00c	0.00c	0.00c	0.00c	0.04b	0.05b	0.12a	0.00c	*	GC–FID, GC–MS
Eugenol	1326	2.15b	2.01b	2.57b	2.33b	2.32b	2.10b	2.55b	3.21a	2.47b	*	GC–FID, GC–MS
β-Damascenone	1359	0.65a	0.54a	0.03a	0.09a	0.05a	0.12a	0.23a	0.24a	0.08a	**	GC–MS
Methyleugenol	1408	0.75bc	1.74a	0.69bc	0.68bc	0.29cd	0.45bcd	0.87b	0.00e	0.58bc	*	GC–FID, GC–MS
Caryophyllene	1414	0.26a	0.02b	0.0b	0.18ab	0.05b	0.67a	0.01b	0.01b	0.06b	**	GC–MS
(*E*)-β-Famesene	1458	0.12ab	0.04ab	0.03b	0.12ab	0.06ab	0.07ab	0.16a	0.05ab	0.07ab	*	GC–MS
4,6-di-tert-Butyl-m-cresol	1462	0.05a	0.06a	0.09a	0.04a	0.02a	0.02a	0.08a	0.03a	0.04a	ns	GC–MS
(*Z*,*E*)-α-Farnesene	1480	0.36a	0.05b	0.01b	0.03b	0.01b	0.02b	0.28a	0.07b	0.02b	**	GC–MS
β-ionone	1490	1.93ab	2.74a	0.29cd	0.11d	0.24d	0.22d	0.08d	1.48bc	0.45cd	**	GC–FID, GC–MS
α-Farnesene	1494	6.08a	0.33ab	0.05b	0.07b	0.05b	0.03b	12.81a	2.36a	0.30ab	*	GC–MS
α-Copaen-11-ol	1582	0.03b	0.03b	0.01b	0.01b	0.01b	0.01b	0.15a	0.13a	0.17a	***	GC–MS
(+)-Carotol	1652	0.04a	0.04a	0.01a	0.01a	0.01a	0.01a	0.03a	0.02a	0.01a	ns	GC–MS
2,3-Dihydrofarnesol	1696	0.05d	0.04d	0.07cd	0.25b	0.07cd	0.08cd	0.44a	0.12bcd	0.21bc	***	GC–MS
Farnesol	1702	1.6bc	0.8c	0.6c	3.2a	0.9c	0.8c	3.2ab	1.2c	1.9abc	***	GC–MS
Farnesal	1552	0.45a	0.48a	0.23a	0.36a	0.15a	0.21a	0.71a	0.32a	0.41a	ns	GC–MS
**Other compounds**												
2-Methylthiolane	952	11.4b	45.8a	0.0d	20.1ab	33.5a	0.0d	10.7b	8.6bc	0.0d	*	GC–MS
1,3,3-Triethoxypropane	1076	12.1ab	20.4a	3.1b	8.4ab	5.5b	6.6ab	0.0b	11.1ab	5.8b	**	GC–MS
Benzothiazole	1186	0.0a	0.0a	10.3a	31.2a	9.1a	0.0a	0.0b	0.0b	0.0b	*	GC–MS

Same letters next to mean values within rows indicate the lack of statistically significant differences at *p* < 0.05, *n* = 5, *ns*—not significant; 0.001 ‘***’; 0.01 ‘**’; 0.05 ‘*’’; ^1^ LRI—linear retention index; the amount of components was determined semi-quantitatively by measuring the relative peak area of each identified compound, according to the NIST (National Institute of Standards and Technology) database, in relation to that of the internal standard. Abbreviations: SF—spontaneous fermentation, SC—fermentation with *Saccharomyces cerevisiae* (Ethanol Red), SB—fermentation with *Saccharomyces bayanus* (cider yeast).

## Supplementary Materials

The following are available online. Table S1: Pearson correlation coefficients between the intensities of the descriptors and the overall note of the brandies obtained from apple musts fermented with different microorganisms. Summary of statistical analyses carried out for results of sensory analysis.
