# Supplementary material for: Saccharomyces bayanus Enhances Volatile Profile of Apple Brandies
Source: Molecules. 2020 Jul 8;25(14):3127. doi: 10.3390/molecules25143127 (PMC7397190; doi:10.3390/molecules25143127)
Supplement: Supplementary file 1 [file molecules-25-03127-s001.pdf]

molecules

MDPI

Saccharomyces bayanus Enhances Volatile Profile of Apple Brandies

Magdalena Januszek [1,*], Paweł Satora [1], Łukasz Wajda [2], and Tomasz Tarko [1]

[1] Department of Fermentation Technology and Microbiology, Faculty of Food Technology, University of Agriculture in Krakow, Balicka Street 122, 30-149 Kraków, Poland; pawel.satora@urk.edu.pl (P.S.); tomasz.tarko@urk.edu.pl (T.T.)
[2] CDC Poland Sp. z o.o., ul. Zagnańska 153, 25-563 Kielce, Poland, lukasz.wajda8@gmail.com
* Correspondence: magdalena.kostrz@urk.edu.pl

Table 1. Pearson correlation coefficients between the intensities of the descriptors and the overall note of the brandies obtained from apple musts fermented with different microorganisms. Summary of statistical analyses carried out for results of sensory analysis.

		Descriptors							
		**Floral**	**Sweet**	**Grassy**	**Fruity**	**Smoked**	**Citrus**	**Pungent**	**Yeast**
**ELISE**	**Spontaneous fermentation**	0.33	0.56	0.00	0.60	0.00	0.82	−0.45	−0.60
	**Ethanol RED (*S.cerevisiae*)**	0.61	0.25	0.50	0.25	0.00	0.00	0.76	0.00
	**Cider Yeast (*S.bayanus*)**	0.67	0.56	0.17	0.17	0.00	0.00	−0.67	0.00
**RUBIN**	**Spontaneous fermentation**	0.60	0.60	0.60	0.33	0.00	0.82	−0.50	−0.17
	**Ethanol RED (*S.cerevisiae*)**	0.17	0.27	0.11	0.56	0.00	0.11	−0.11	0.00
	**Cider Yeast (*S.bayanus*)**	0.65	0.53	0.00	0.36	0.00	0.80	0.52	0.00
**TOPAZ**	**Spontaneous fermentation**	0.00	0.48	0.00	0.50	0.00	0.44	−0.74	0.00
	**Ethanol RED (*S.cerevisiae*)**	0.33	0.19	0.50	0.60	0.00	0.60	−0.18	0.00
	**Cider Yeast (*S.bayanus*)**	0.50	0.56	0.00	0.22	0.00	0.17	−0.38	0.00
**Significance level**		***	***	***	***	ns	***	*	***

The same letters next to mean values within columns indicate the lack of statistically significant differences at $p < 0.05$; *ns* not significant; 0.001 '***'; 0.01 '**'; 0.05 '*'.
